# Cross cultural evaluation of the Warwick-Edinburgh mental well-being scale (WEMWBS) -a mixed methods study

**DOI:** 10.1186/1477-7525-11-27

**Published:** 2013-02-27

**Authors:** Frances Taggart, Tim Friede, Scott Weich, Aileen Clarke, Mark Johnson, Sarah Stewart-Brown

**Affiliations:** 1 Warwick Medical School, University of Warwick, Coventry, CV4 7AL, UK; 2 Department of Medical Statistics, University Medical Center Göttingen, Göttingen, Germany; 3 Mary Seacole Research Centre, Faculty of Health & Life Sciences, De Montfort University, The Gateway, Leicester, LE1 9BH, UK

**Keywords:** Mental well-being, WEMWBS, Cross cultural, Validation, PROMS (patient reported outcome measures), Ethnicity (MeSH: ethnic groups)

## Abstract

**Background:**

We aimed to validate the Warwick-Edinburgh Mental Well-being Scale (WEMWBS) among English speaking adults representing two of the minority ethnic groups living in the UK, self-identified as Chinese or Pakistani by background, in a mixed methods study.

**Methods:**

Quantitative data were collected in two cities in the West Midlands, UK. Item response, dimensionality, internal consistency, and construct validity of the WEMWBS were assessed in Chinese and Pakistani groups separately, using data from both cities combined.

Qualitative data were collected in the first city in eight focus groups of different ages recruited by the community workers. Three mixed sex Chinese and five single sex Pakistani groups discussed ease of completion and comprehension of items, together with overall reactions to the scale and underlying concept.

Results of quantitative and qualitative analysis were examined for commonalities and differences.

**Results:**

Item completion and item total correlations were satisfactory in both groups. In the Chinese data, Exploratory Factor Analysis showed a single factor with loadings ranging from 0.60 to 0.82 for all 14 items. In the Pakistani data, three factors reached statistical significance; however, a substantial drop in eigenvalues between the first and second factors and the limited variance explained by the second and third factors supported a one-factor model. All items loaded on this factor from 0.51 to 0.83.

In the Chinese and Pakistani data respectively, Cronbach’s alpha was 0.92 (0.89 – 0.94) and 0.91 (0.88 – 0.94); Spearman’s correlation with GHQ-12 was - 0.63 (−0.73 to −0.49) and −0.55 (−0.70 to −0.36), and with the WHO-5 0.62 (0.46-0.75) and 0.64 (0.50 to 0.76).

Qualitative analysis revealed good comprehension and ease of completion of almost all items. Some culturally determined differences in understanding of mental well-being, which varied both between and within communities, emerged.

**Conclusions:**

The WEMWBS was well received by members of both Pakistani and Chinese communities. It showed high levels of consistency and reliability compared with accepted criteria. Data were sufficiently strong to recommend the WEMWBS for use in general population surveys.

## Background

Governments are increasingly recognising the need to improve the mental well-being of their populations [1-3]. As a result, measures are needed that can extend the monitoring of mental health beyond the presence or absence of mental disorder, and be used to evaluate policies and programmes to improve mental well-being. The Warwick Edinburgh Mental Well-being Scale [4] (WEMWBS) was developed to measure mental well-being in the general population and has been validated in student samples in England and Scotland [4], population samples in Scotland [4] and Northern Ireland [5] and among teenagers [6]. It is responsive to change [7] and highly acceptable in both clinical and non-clinical settings [4,6,8].

We sought to assess the extent to which the WEMWBS is suitable for measuring mental well-being in different ethnic groups because concepts and experience of mental health and well-being may vary with culture and beliefs [9-13] and both language and culture may affect responses to measurement scales [14]. It has been said that scales developed particularly in the UK and the USA embody an individualistic view of life and that this may not apply to other cultures [15]. We sought to assess the extent to which the WEMWBS is suitable for measuring mental well-being in different minority ethnic groups.

In England and Wales 8.8% of all households contain at least one family member for whom English is not their main language [16]. The funding available restricted the study to two minority groups. We selected Chinese and Pakistani groups because their beliefs and cultural backgrounds differ both from each other and from the prevailing white majority. People who describe themselves as having a Chinese family background comprise 0.7% of the population in England and Wales, those describing a Pakistani family background comprise 2.0% [16].

In China Confucianism was the dominant value system for many centuries with its central theme of collective welfare, caring for family and acceptance of the hierarchical order of the world. These values take precedence over those of striving for individual happiness. Taoism has also been prevalent in China; this philosophy teaches tranquillity as a route to clarity, and mental well-being is perceived as encompassing an inner sense of balance and contentment with life. Both these value systems have been described among Chinese people resident in the UK [13].

The most common faith among Pakistani people is Islam and 4.8% of UK population report Islamic faith [16]. Islam is a monotheistic religion and Muslims follow the teachings of Muhammad believing that the Qur’an represents the word of God. One study reported that the fulfilment of gender roles and the protection of the family are important religious activities for a Muslim [10]. UK Muslims report that mental distress is understood in the context of religious beliefs. Many older Pakistani people consult traditional healers and religious leaders from their own community and belief in possession by spirits, who may be malevolent, is evident among some members of the Pakistani community [17]. Evidence from a recent survey in the UK suggested that people with a Pakistani background had lower well-being, measured mainly by life satisfaction, than white people while this was not the case for people with Chinese family background [18].

We adopted a mixed methods approach to assess the validity of the WEMWBS and perceptions of mental well-being among English speaking adults who identified themselves as having Chinese or Pakistani heritage. The study was set initially in Birmingham where these groups were well represented and public health professionals at the Primary Care Trust were keen to support the research. It was later extended to Coventry where quantitative but not qualitative data were gathered as part of a general population health survey. This increased the demographic heterogeneity and size of the study population.

## Methods

The WEMWBS is a 14 item scale designed to measure positive mental health or mental well-being. It comprises both hedonic elements (happiness, joy, contentment) and eudaimonic elements (psychological functioning, autonomy, positive relationships with others, sense of purpose in life). We undertook quantitative surveys in purposive samples from two cities and focus group discussions in age- (and in one ethnic group, sex-) specific groups of adults who self-defined as English speaking.

### Quantitative validation

#### Questionnaires

Birmingham sample: A booklet containing the WEMWBS, two comparator scales – the twelve item General Health Questionnaire (GHQ-12) [19] and the World Health Organisation Well-being 5 questionnaire (WHO-5) [20,21] and a demographic questionnaire was designed for this study at the University of Warwick and approved by the Chinese and Pakistani community representatives and Primary Care Trust (PCT) representatives in Birmingham.

Coventry sample: A questionnaire suitable for a general health survey (available from the authors) was designed by Coventry PCT in collaboration with the University of Warwick, this covered a range of health indicators and demographic details including the WEMWBS, but not the GHQ-12 or WHO-5. The Coventry survey containing the WEMWBS was an interview based questionnaire delivered door to door but the WEMWBS part of it was self-completed while the interviewer waited. Some interviews were conducted in the street.

#### Participants and data collection

As recruitment in minority communities can be very challenging, specific strategies were identified in collaboration with the local PCTs, namely those in Birmingham East and North, and Coventry.

In Birmingham, community workers used their links with and knowledge of the local communities to approach adults who self-identified as belonging to one of these two ethnic groups during a three month period, December 2009 to February 2010. Participants were aged 16 to 75 years and quota sampling ensured that men and women were equally represented. In the Chinese community potential participants were approached at a variety of locations including Chinese restaurants and supermarkets and a local University and College. The Pakistani group were approached in single sex fitness gyms (popular gathering places especially for women as access was provided free of charge to local residents), by door-knocking in high density streets and by word of mouth using social networks. Participants were provided with written information about the study and given the option of answering the questionnaire on the spot or taking it away to complete at home and return in a prepaid envelope. Most chose the first option. Participants were given a £5 ASDA voucher as a gift when they returned their completed questionnaire.

In Coventry, participants aged 16–75 years were identified during the Coventry Household Survey [16] for which households were selected on a postcode basis across all 42 MSOAs (Middle Super Output Areas containing approximately 6,500 households). Data were collected by means of face to face interviews in the home (94%) or on the street (less than 6%) during winter 2009/10. The WEMWBS section of the questionnaires was self-completed at the time of interview either at the door or in the street. We selected all respondents who described their ethnicity as Pakistani or Chinese.

#### Psychometric analyses

Coventry and Birmingham data were combined for all analysis for which data were available in the Coventry sample. Chinese and Pakistani samples were analysed separately. Data were examined for normality, floor and ceiling effects, and missing and popular responses.

Dimensionality was explored in exploratory factor analysis using the principal components method. The number of factors was determined by examining scree plots for the last substantial drop in magnitude of the eigenvalues [22]. Cronbach’s alpha was used to assess internal consistency with values above 0.8 indicating desirable levels [11]. Spearman’s rank correlation coefficients were computed for each of the 14 items with the total of the remaining 13 items and reported with 95% confidence intervals (CI) obtained by nonparametric bootstrap with 9999 bootstrap replications [23].

In order to assess construct validity (external consistency) correlations between the WEMWBS, the GHQ-12 and WHO-5 were computed using Spearman’s correlation coefficients in the Birmingham samples. Nonparametric bootstrap confidence intervals and p-values of approximate significance tests using the null hypothesis of zero correlation were calculated. All analyses were carried out using SAS 9.3 statistical package.

#### Sample size calculation

With high communalities, which could reasonably be expected since unidimensionality of the WEMWBS had been demonstrated in other samples [4,7], sample sizes of about 100 are sufficient to carry out an exploratory factor analysis [24]. A sample size of 100 participants gives a 95% confidence interval for the correlation coefficient no wider than 0.3 as long as the correlation is larger than 0.5 or smaller than −0.5, which was considered sufficiently precise for our purposes.

### Qualitative evaluation

#### Participants and data collection

Qualitative evaluation was undertaken in Birmingham. The same white, female interviewer (FT) led all of the focus groups. The female Chinese community worker who recruited the groups was present for all of the Chinese focus groups. The male Pakistani community worker was present for all the Pakistani male groups and another young Pakistani woman was present for all the Pakistani women’s groups. This approach worked pragmatically and enabled us to gather the views of members as well as representatives of the community. Having a researcher who was not a member of the community may have encouraged participants to explain things which might seem obvious to someone of their own culture. Since the community workers who recruited participants were employed by the PCT in Birmingham the study may also have been seen as sanctioned by the National Health Service (NHS). No participants who had been included in the quantitative survey were included in qualitative interviews.

Potential participants were informed about the study by the community workers and given a letter of invitation, a copy of the information sheet and consent forms one week before the focus group. Ten of those agreeing to take part were invited to each group and completed a questionnaire booklet designed to record demographic data. Participants who attended the focus groups were given £20 to cover travelling expenses, childcare or other expenses incurred in attending the group.

Chinese participants were included in one of three mixed sex groups according to age: people aged 16 to 24 recruited at college and university (Aston), people age 25 to 49 recruited from among community workers’ friends and contacts, mostly from professional backgrounds, and people age 50 to 75 recruited from residents and family members at a community centre and a housing association home.

We held single sex focus groups for Pakistani adults since we were advised by the community worker that attendance would be better and women’s views more likely to be heard. Five single-sex Pakistani focus groups were held among the following groups: young men aged 16 to 24 years recruited from a local youth project, men age 25 to 49 years recruited at a Fitness Gym, older men aged 50 to 75 years recruited through a taxi base and men’s social groups, women aged 16 to 24 years recruited through college and young women’s groups, women aged 25 to 49 years recruited through sewing classes, Fitness Gyms and word of mouth. We were unable to recruit to an older women’s group.

Focus groups were held in community centres and meeting places commonly used by the local community [25], for example a room in a Pakistani restaurant and the day room in a Chinese housing association building.

The topic guide was designed to identify whether participants found the WEMWBS items easy to complete and whether they understood them in the same way as the general UK population. It also aimed to investigate the acceptability of the entire scale and the extent to which this covered concepts of mental well-being relevant to each community. The protocol, topic guide and other study materials such as questionnaires and information sheets are available as supplementary data from the authors.

### Analysis

Focus group discussions were recorded and transcribed in full and comments analysed using NVivo. Responses relating to items were analysed item by item, discussion relating to concepts of mental well-being was analysed by themes.

### Comparison of qualitative and quantitative findings

Results of quantitative and qualitative analyses were examined for commonalities and differences.

### Ethics approval

The Biomedical Research Ethics Committee at Warwick Medical School approved both quantitative and qualitative components of the study.

## Results

### Quantitative evaluation

#### Response rate

240 questionnaires were distributed in Birmingham, 120 each to the Chinese and Pakistani groups. Many people who were approached declined to complete the questionnaire. Of those who agreed to take a questionnaire 113 (94%) were returned for the Chinese group and 108 (90%) for the Pakistani group.

In the Coventry Household Survey participants who had described their ethnicity as Chinese or Pakistani were selected from all respondents. The number of people interviewed overall in Coventry was approximately half of the number of households selected for the survey (3552 interviews among approximately 6,500 households). There were 41 Chinese participants and 103 Pakistani participants, numbers proportionate to the Chinese (1.2%) and Pakistani (3.0%) populations in Coventry. None of the Chinese respondents were interviewed on the street but 16 of the Pakistani respondents (15.5%) were.

#### Participant characteristics

All participants who answered all items in the WEMWBS were included for analysis while incomplete responders were excluded. Thus 152 of the 154 Chinese respondents and 183 of the 211 Pakistani respondents were included. Demographic descriptors of the Coventry and Birmingham samples are shown in Additional file 1: Tables S1a and S1b respectively. For psychometric analysis the Coventry and Birmingham samples were merged.

The median age of participants who completed the WEMWBS in the Chinese sample was 29 years, ranging from 18–90 years. There were 89 men (59%) and 63 women (41%). Among men, 71 (80%) were in paid work or full time education and for women this figure was 53 (84%). There were differences in demographics between the two cities. The Coventry sample mostly comprised young students (71% students) with more men than women. The Birmingham group were more likely to be in the 25–49 year age group and in paid work (69% in paid work).

The median age of participants who completed the WEMWBS in the Pakistani sample was also 29 years (range 17–71 years). There were 108 men (59%) and 74 women (40%) and one person of unknown gender. Among men 73 (68%) were in paid work or full time education while for women this figure was 22 (30%). As with the Chinese group there were more men than women in the Coventry sample, with 57 (70%) men. There were differences in mean WEMWBS scores between the two cities in the Pakistani sample: Coventry: 52.0 (50.1 – 53.9), Birmingham: 47.7 (46.0 – 49.5) (Supplementary data S1a-b).

#### Missing and popular responses

The frequency of complete responses was 152/154 (99%) among the Chinese and 183/211 (87%) in the Pakistani group. Numbers of incomplete responders were too small to identify demographic characteristics for the Chinese group (only two people both from Birmingham sample).

Among the 28 people in the Pakistani sample who did not complete the questionnaire in full 21 (75%) were from the Coventry sample. There were no differences in age or gender between the incomplete responders and the complete responders but there was a difference in work status. The incomplete responders were less likely to be in paid work or studying (Pearson’s Chi2 = 11.8, p < 0.003). The item most likely to be missing among Pakistani responders was item 3 “I’ve been feeling relaxed”.

#### Normality and floor and ceiling effects

**Figure 1:**
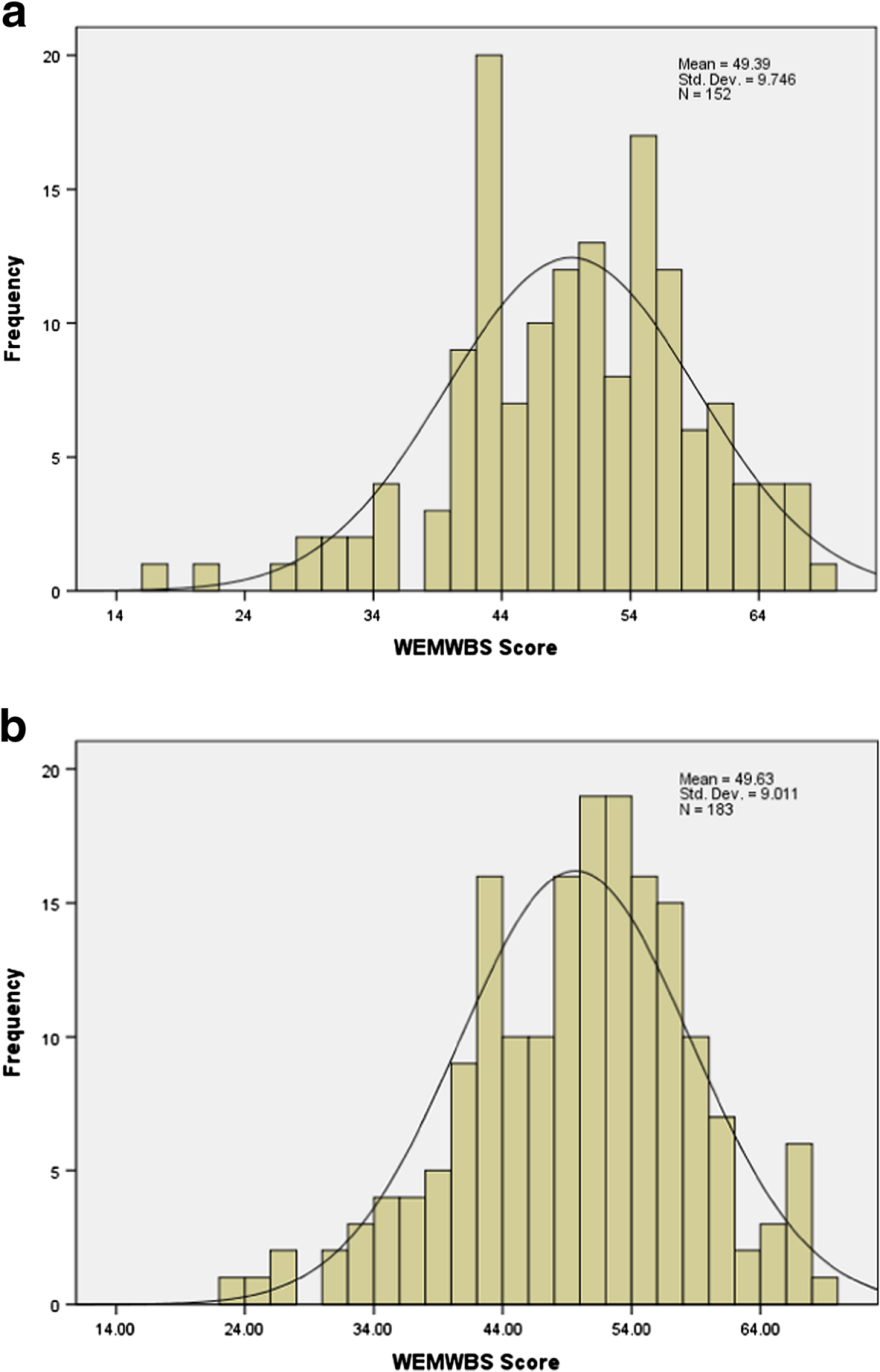
a. Score distribution for WEMWBS in Chinese Samples (Birmingham and Coventry combined). b. Score distribution for WEMWBS in Pakistani Samples (Birmingham and Coventry combined).

The distributions of responses from complete responders are shown in Figures 1a and b. For the Chinese sample the distribution of total scores was normal with a slight tail towards the lower end (skew = − 0.434, p < 0.05), the mean score was 49.39 (95% CI 47.83 to 50.95) and the median 50. For the Pakistani sample the total scores were normally distributed with a mean score of 49.63 (95% CI 48.32 – 50.94) and a median of 51. These median scores are similar to a median score of 51 in the population sample in Scotland reported by Tennant et al. [4] and median score 50 in a population sample in Northern Ireland reported by Lloyd & Devine [5]. There was no evidence of floor or ceiling effects in either sample. A slight negative skew was also seen in the Tennant study.

#### Internal consistency

Cronbach’s alpha was 0.92 (95% CI 0.89 – 0.94, n = 152) and 0.91 (95% CI 0.88 – 0.94, n = 183) respectively for the Chinese and Pakistani samples. These fall well above the recommended lower limit of 0.8 [11]. The figure reported by Tennant et al. was 0.91 for the population sample [4], that reported by Lloyd & Devine was 0.93 [5]. Our findings for both groups are thus consistent with previous studies in other population samples.

#### Item total correlations

Table 1 shows Spearman rank correlation coefficients for each item with the total of the remaining items. For the Chinese group the item total correlations ranged from r = 0.51 for item 4 (I’ve been feeling interested in other people) to 0.76 for item 10 (I’ve been feeling confident).

**Table 1:** Item – total correlations

	**Chinese sample**				**Pakistani sample**			
**Item**	**Correlation**	*Lower limit	*Upper limit	**p-value**	**Correlation**	*Lower limit	*Upper limit	**p-value**
wemwbs_1	0.61	0.48	0.72	<0.001	0.28	0.06	0.49	0.003
wemwbs_2	0.70	0.60	0.78	<0.001	0.68	0.54	0.79	<0.001
wemwbs_3	0.57	0.44	0.68	<0.001	0.62	0.48	0.74	<0.001
wemwbs_4	0.51	0.36	0.64	<0.001	0.51	0.34	0.65	<0.001
wemwbs_5	0.54	0.41	0.66	<0.001	0.55	0.38	0.68	<0.001
wemwbs_6	0.64	0.51	0.74	<0.001	0.72	0.59	0.81	<0.001
wemwbs_7	0.67	0.56	0.76	<0.001	0.65	0.51	0.77	<0.001
wemwbs_8	0.75	0.67	0.83	<0.001	0.74	0.62	0.83	<0.001
wemwbs_9	0.60	0.48	0.70	<0.001	0.64	0.48	0.77	<0.001
wemwbs_10	0.76	0.68	0.82	<0.001	0.76	0.65	0.84	<0.001
wemwbs_11	0.67	0.57	0.76	<0.001	0.61	0.46	0.73	<0.001
wemwbs_12	0.68	0.57	0.76	<0.001	0.65	0.48	0.80	<0.001
wemwbs_13	0.57	0.43	0.68	<0.001	0.69	0.55	0.81	<0.001
wemwbs_14	0.71	0.61	0.80	<0.001	0.73	0.58	0.84	<0.001

*95% Confidence Interval.

For the Pakistani group, item total correlations ranged from 0.28 for item 1 (I’ve been feeling optimistic about the future) to 0.76 for item 10 (I’ve been feeling confident) with item 4 also correlating at 0.51. These findings are comparable with the findings of the Tennant population sample where the range was from r = 0.5 to r = 0.75 with the possible exception of the low figure for item 1 in the Pakistani group.

#### Dimensionality – Factor analysis

For the Chinese sample only one factor was significant (Table 2); this factor explained 52% of the variance. Item loadings ranged from 0.60 for item 4 (interested in other people) to 0.82 for item 8 (feeling good about myself) (Table 2). The eigenvalue for this factor was 7.27. There were no other factors with eigenvalues greater than 1.

**Table 2:** Factor pattern

**Chinese**		**Factor1**	**Pakistani**	**Factor1**	**Factor2**	**Factor3**
**WEMWBS_1**	I’ve been feeling optimistic about the future	**0.66930**	I’ve been feeling optimistic about the future	**0.50982**	**0.61756**	−0.19523
**WEMWBS_2**	I’ve been feeling useful	**0.75723**	I’ve been feeling useful	**0.63094**	0.37766	−0.08334
**WEMWBS_3**	I’ve been feeling relaxed	**0.63469**	I’ve been feeling relaxed	**0.65596**	−0.37348	−0.12848
**WEMWBS_4**	I’ve been feeling interested in other people	**0.59770**	I’ve been feeling interested in other people	**0.52642**	0.27406	**0.45692**
**WEMWBS_5**	I’ve had energy to spare	**0.61365**	I’ve had energy to spare	**0.56372**	**−0.47860**	−0.06849
**WEMWBS_6**	I’ve been dealing with problems well	**0.71795**	I’ve been dealing with problems well	**0.74168**	0.09701	−0.16827
**WEMWBS_7**	I’ve been thinking clearly	**0.78009**	I’ve been thinking clearly	**0.80254**	−0.02221	−0.29462
**WEMWBS_8**	I’ve been feeling good about myself	**0.82139**	I’ve been feeling good about myself	**0.83030**	−0.12068	−0.06225
**WEMWBS_9**	I’ve been feeling close to other people	**0.67591**	I’ve been feeling close to other people	**0.66770**	0.17214	0.41795
**WEMWBS_10**	I’ve been feeling confident	**0.80932**	I’ve been feeling confident	**0.79350**	−0.00647	−0.21192
**WEMWBS_11**	I’ve been able to make up my own mind about things	**0.75827**	I’ve been able to make up my own mind about things	**0.70610**	0.10874	−0.32483
**WEMWBS_12**	I’ve been feeling loved	**0.74342**	I’ve been feeling loved	**0.71652**	−0.00825	0.41039
**WEMWBS_13**	I’ve been interested in new things	**0.66411**	I’ve been interested in new things	**0.70674**	−0.22895	0.35526
**WEMWBS_14**	I’ve been feeling cheerful	**0.79649**	I’ve been feeling cheerful	**0.80703**	−0.19257	0.02569

For the Pakistani sample three factors were significant, explaining 48%, 8% and 7% of the variance respectively (Table 2). The first eigenvalue was 6.8 dropping to 1.1 for the second and 1.0 for the third. Factor loadings on the first factor ranged from 0.51 for item 1 (I’ve been feeling optimistic about the future) to 0.83 for item 8 (I’ve been feeling good about myself). The findings are also consistent with those in other study populations. Principal component analysis in the Northern Ireland sample gave component loadings ranging from 0.56 for item 1 (I’ve been feeling optimistic about the future) to 0.86 (I’ve been feeling confident) [5].

#### Construct validity – External consistency

Spearman’s correlation for the WEMWBS with other scales (data available in Birmingham samples only) was as follows:- Chinese with the GHQ-12: -0.63 (95% CI −0.73 to −0.49, p < .0001, n = 108), and with the WHO-5: 0.62 (95% CI 0.46-0.75, p < .001, n = 111), Pakistani with the GHQ12: -0.55 (95% CI −0.70 to −0.36, p < 0.001, n = 96) and with the WHO-5: 0.64 (95% CI 0.50 to 0.76, p < 0.001, n = 101). These are consistent with correlations demonstrated in the population sample in the Tennant study, where the correlation of the WEMWBS with the GHQ-12 was 0.53 (the WHO-5 was not used in this study) [4].

### Qualitative evaluation

Twenty-two Chinese and 47 Pakistani adults took part in the focus group discussions. Twelve Chinese and 43 Pakistani participants lived in post code areas marked with indices of multiple deprivation that were in the top (most deprived) 10% in England, 3 and 18 respectively were not in paid work or studying, and 3 and 9 respectively left school at age 15 years or less (Additional file 1: Table S2). The focus groups were conducted in English, but in the event one older Chinese woman and three middle aged Pakistani women were not fluent in English and translation was undertaken for these participants by the community worker in the former group and other young women participants in the latter. The Chinese woman spoke Cantonese. Occasionally in the Pakistani groups, other participants spoke in their native language and the community worker translated. The native language of the young and middle age Pakistani women was Pashtun. For the middle aged male group it was also Pashtun with the exception of one person who spoke Mirpuri. For the young men and the older men their language was a mixture of Urdu, Pashtun and other Pakistani languages.

#### Focus group discussion relating to items

All items were thought to be easy to understand with the possible exception of item 1 (optimism) among the Pakistani women. One of the younger women in the middle age group informed us that there was no translation for the word ‘optimistic’ in Pashtun. Although most in the group felt they understood the word, some understood it as ‘happy for the future’ and it was not clear that the expectation that things would work out well was understood.

One item was thought to have potential for misinterpretation. Young men, in both Chinese and Pakistani groups interpreted the item ‘feeling interested in other people’ in a sexual context.

*‘Yeah man, girls!* [YPM1]

Although all participants found the items easy to understand one young Pakistani man was concerned that others might not understand the items because they were not used to personal reflection. He gave this example.

*‘I know a lot of people in my college ………. they’d struggle to understand. …it’d just be like I’m interested in new things. …I want to go out and get new trainers… You know’* [YPM2]

Questions which required generalisation across settings, particularly feeling relaxed and useful, were considered difficult to answer by some participants.

*“This question is a bit vague, as in you mean useful at work? Useful at home?”* [MPM1]

#### Discussion relating to the scale and coverage of concepts of well-being

Some participants observed that the WEMWBS was good to complete because it made you think about your life in terms of mental well-being. Participants in both ethnic groups talked of the importance of interpersonal relationships for mental well-being. Both groups also discussed concepts relevant to mental well-being that were not covered by the scale.

Chinese participants in general espoused an internal model of mental health, believing that it depended on your own actions and attitudes. This belief came over very strongly in all three age groups, which also encompassed people from a range of socioeconomic circumstances. The youngest group in particular expressed the view that it was unfair to burden others with your problems and that mental resilience and strength was a virtue.

*It’s how you think really isn’t it? And how positive you are in life affects your well-being.* [YCW1]

*Your life is in your own hands. It’s down to you. You don’t blame other people for your life.* [YCW2]

The Chinese tended to be dismissive of depression believing that it was over diagnosed in England.

Both groups expressed the belief that hardship was to be expected. Some went as far as to say that suffering was character building. The middle Chinese group (aged 25–49), who were mostly professionals, pointed to the importance of physical activity for mental health saying that going to the gym would give you more energy when you were feeling tired.

Most of the Chinese group was atheist, but there were two Christians. The Christians interpreted happiness as an expression of love. They said that happiness was reciprocal, in the sense that love generates love and happiness in the person who is loved.

Pakistani participants espoused a more social model of mental health. Men talked of depression in middle aged women born in Pakistan who took many years to adjust to living in England. They saw worries about the family and financial problems as the major cause of mental suffering. Men also talked about anger and frustration arising from unemployment and the problems that unemployment caused within families where the father was the only breadwinner.

*But when wife got nothing – I’m not working – I’m at home yeah – She shouts at me. She shout at me that I’m here. What you doing at home? What should I do? I’m finding job I can’t find a job… .* [OPM1]

They also spoke of not being able to admit to mental health problems.

*You know like we’re supposed to be like these warrior people. If you do show signs of mental illness [..] it will show a weakness saying you are not all a man.* [MPM1]

The item “I’ve been able to make up my own mind about things” provoked discussion in all Pakistani groups, but attitudes to autonomy and its importance for mental well-being varied. The item was perceived to implicitly value individualism, which was not universally considered a good thing. Some of the women believed that decisions, other than the most trivial, should be made with the advice of family, close friends and relatives.

“In this community you ask your elders for advice” [MPW1]

*“You like other people’s opinions … family members – you’d ask them am I making the right decision?”* [MPW2]

Some men gave a more considered view.

*“I think of it from different sides. If you’re a bit arrogant OK then … Could be arrogance ...... But then if you’re completely indecisive then that would be a mental problem”.* [MPM2]

On the other hand, many young men and women wished to have more freedom in important decisions such as marriage (men) and going to university to study (women) than the family and community allowed. One young man felt it was important to be able to make your own decisions,

*“You can’t let people walk all over you. For example you know living your life for you I mean. You’ve got your own life haven’t you? ................. You need to be your own individual and make your own decisions.”* [YPM2]

Some young women felt able to make their own decisions while others felt they were not allowed to and others still did not feel capable of making them.

*“I’ve no choice*”. [YPW3]

*“I want to look to others quite often for advice…… I can’t make up my mind on my own”.* [YPW4]

FT So do you think that’s a good thing or would you like to be more able to make up your own mind?

*“Your confidence…”.* [YPW5]

*“Yeah I think I’ll always be like that”* [YPW4 …]

Conflict between western and traditional ways of life in which careers and lifestyle choices were arranged for sons and daughters was considered detrimental to mental well-being.

Older Pakistani women stressed the importance of a Pashtun word which roughly translated as ‘responsibility’. Both men and women in the Pakistani groups mentioned the spiritual interpretation of mental well-being in their community and the practice of consulting local religious leaders as well as respected family members about problems. Some espoused the Islamic view that prayer five times daily would maintain good mental health while for others this was seen as a temporary solution which could not solve problems such as unemployment.

### Comparison in findings between quantitative and qualitative findings

These qualitative findings were consistent with and elaborated on some of the findings from the quantitative analysis. The items with the highest item-total correlation and strongest factor loadings in both groups were ‘feeling good about myself’ and ‘feeling confident’. Neither of these items were a cause for comment in any of these focus groups either about comprehension or relevance to mental well-being. Responses to item 1 (feeling optimistic about the future) had the lowest item total correlation and factor loading for the Pakistani group and in focus groups the reason for this became clear. There is apparently no translation for “optimistic” in Pashtun and a quarter of Pakistanis completing the questionnaire were Pashtun speaking and born abroad. This question was also difficult for English teenagers in a previous study many of whom did not understand the word optimistic [6].

Among the Chinese group the item with the lowest item total correlation and factor loading was ‘I have been feeling interested in other people’. This was also the second lowest item correlation and factor loading in the Pakistani group. “I’ve been feeling interested in other people” was misunderstood by several young and middle aged Pakistani men and one Chinese man who saw a sexual interpretation for this question in common with the UK teenage group in a previous study [6].

## Discussion

We chose to study the WEMWBS in English speaking people who identified themselves as belonging to two diverse minority ethnic groups and were living in the UK in order to evaluate the psychometric properties of the scale and discover how the scale was viewed in these very different cultural groups. The samples with which we conducted the study were selected pragmatically with a view to hearing the voices of as wide a selection of the community as possible, particularly those who are often ‘hard to access’. We were dependent on community workers for achieving this and needed to trust their expertise, but without them we would have been unlikely to succeed. The demography of the sample suggests that they did succeed to a reasonable extent. Quota sampling in Birmingham ensured a good representation of both men and women in both qualitative and quantitative evaluations, whereas the door to door approach in Coventry resulted in a higher proportion of responses from men than women. The Pakistani sample from both cities was deprived, as is the Pakistani population in general in these cities. A high proportion of the Coventry Chinese sample were students as would be expected from population demographics. Fitness gyms were used for recruitment in Birmingham because at this time these gyms were provided on an open access basis as a public health intervention and people with obesity and diabetes were encouraged to attend. The gyms also provided an acceptable social outlet and meeting place for women in particular who had few other opportunities to socialise outside the home. Although it is possible that people with diabetes or who were obese were over-represented we believe we recruited a sample which could provide a good estimate of the performance of the measure in these minority groups. The slightly lower scores for the WEMWBS among the Birmingham Pakistani sample compared to the Birmingham Chinese sample are consistent with the findings of earlier studies showing greater levels of anxiety and depression among people of Pakistani background in the UK when compared with the majority population [18], [26]. This difference was not seen in the smaller Coventry sample. Most of the incomplete responses by the Pakistani sample were from residents in Coventry. The association of incomplete response with not being in paid work or studying suggests that comprehension of English could have been a problem in this sample. No specific explanation was offered with regard to the WEMWBS in the Coventry survey while the information sheet may have facilitated complete response in the Birmingham sample.

To our knowledge this is the first study to attempt this level of validation of a mental well-being scale among minority ethnic groups. One strength of the study is that we have accessed some of the most deprived people living in two UK cities and have gathered mixed methods data from two very different communities. The use of gender specific Pakistani groups and splitting participants into different age groups in both communities meant that we were able to identify some conflicting attitudes and beliefs within communities. Another strength was that the qualitative and quantitative findings of this study are remarkably consistent. They are also consistent with findings of other validation studies among adults in the UK [4], [5] and 13 to 16 year old teenagers [6]. In the latter group the items which performed less well were the same two items ‘optimism’ and ‘interest in other people’. The item about optimism was also the one that had the lowest loading study in the study in Northern Ireland [5].

We have shown that the WEMWBS performs very well in the Chinese community from a psychometric perspective. The scale was well received in all focus groups and all the items were endorsed as appropriate for mental well-being. The qualitative data pointed to the possibility of misinterpretation of one item among some members of the community. This item ‘I’ve been feeling interested in other people’ has caused discussion in other evaluations of the scale [5]. These data also revealed aspects of mental well-being considered relevant in this community which are not covered in the scale. The most important of these was an attitude of self-reliance and self-responsibility for mental health. It was considered important not to burden others with personal woes. Depression was seen as weakness and the capacity to endure hardship as necessary for well-being. Espousing a positive attitude to life and taking exercise to improve energy levels were also mentioned as important. Spiritual components of well-being were not mentioned in the Chinese groups where very few members belonged to religious denominations, but the generative nature of love as important for own and others well-being was mentioned in one person who was a Christian.

The psychometric properties of WEMWBS were not as clear-cut among the Pakistani community with a suggestion of multidimensionality emerging from the exploratory factor analysis. Other aspects of these analyses, however, clearly pointed to one main factor suggesting that the performance of the scale is likely to be good enough to capture the mental well-being of the Pakistani community in general population surveys. The Pakistani groups identified difficulties in answering an additional item ‘feeling optimistic about the future’ particularly Pashtun speakers which may be the reason the psychometric properties of WEMWBS were not as strong in this group as in the Chinese. This item also raised issues in qualitative discussion of the WEMWBS among teenagers [5]. It is possible that those who focus more on the here and now, the young and those who espouse some Eastern spiritual traditions, are more prepared to let the future take care of itself than adults with a Western orientation. The item ‘I’ve been feeling interested in other people’ also raised some questions in this group. With regard to the extent to which concepts of well-being in the Pakistani groups matched onto the WEMWBS items, the item which caused most discussion was ‘I’ve been able to make up my own mind about things’. It is clear that there are differences within the Pakistani community with regard to attitudes towards the relative importance of individual autonomy and fitting in with social expectations and that these differences were a source of conflict and mental health problems within the community. Pakistani groups also identified prayer and spirituality as important to well-being as well as the concept of responsibility. Neither of these two aspects of well-being are represented in the WEWMBS. Recent research on mental well-being in Singapore where there are three ethnic groups Chinese, Malay and Indian (both Muslim and Hindu), identified the need for coverage of spiritual and religious practices in mental well-being instruments [27]. It is notable that in this study, in which a new instrument was developed on the basis of qualitative assessment of concepts of well-being and compared to many existing instruments, SWEMWBS (the shortened version of the WEMWBS) correlated with the new scale better than other instruments on all subscales except spirituality. This suggests that the WEMWBS does represent other aspects of mental well-being well, even in these very different cultural groups.

Culturally influenced views of mental well-being may, however, potentially enrich understanding of this concept among the majority population. In future, scales incorporating aspects of mental well-being such as spirituality and personal responsibility for health could arguably be incorporated into scales of mental well-being and validated across all cultural groups.

An earlier report of mental well-being among ethnic minorities in the UK concluded that what constitutes mental well-being is different in different cultures [12], but overall we identified more consensus than disparity in concepts of well-being in these minority communities compared to the UK general population. The conclusion of the earlier study was based on theoretical analysis of philosophical differences and focus groups with community representatives. The conclusions may or not apply to individuals within the community.

One limitation of this study was that we did not translate the WEMWBS so the population necessarily comprised people who spoke English and who elected to answer the questionnaire. The key question we wanted to answer was whether the WEMWBS would provide valid data on mental well-being in minority communities in general population surveys, which in the UK are mostly completed in English. A question still remains whether the WEMWBS is acceptable and effective for the part of the population who do not speak English. Translated questionnaires have had a variable amount of success in their validity among minority ethnic groups. Some, admittedly for measurement of physical symptoms rather than mental well-being have found a loss in sensitivity and specificity [28].

Cultural differences may be diminished in communities who have assimilated English language and cultural influences. The varied views on the item about making up your own mind among Pakistani focus groups may be evidence of this. In a study conducted more than 20 years ago non-English speaking Pashtun mothers living in Britain believed both happiness and unhappiness were sent by God. The important thing was not individual suffering but the maintenance of correct social and religious behaviour in the face of it [29]. These views were not expressed in any of our focus groups but the value of prayer was mentioned in some. It is to be expected that beliefs and expectations of life may change over time and place among all cultures. It is notable, however, that the psychometric properties of the WEMWBS remained robust in the face of the inter- and intra-cultural diversity we identified in the qualitative part of this study, suggesting that the scale is suitable for measuring mental well-being in these different ethnic groups.

## Conclusions

The WEMWBS was well received by English-speaking members of both Pakistani and Chinese communities and showed high levels of consistency and reliability when compared with accepted criteria. These findings suggest that the WEMWBS is acceptable across different cultural groups and sufficiently sound from a psychometric perspective to be valid in general population surveys. Culturally determined views of mental well-being amongst some members of these minority ethnic communities and variability within ethnic groups indicates that cultural diversity should be accommodated in well-being scales designed to be used across populations. Further research is needed to develop scales in the languages of these two minority ethnic groups.

## Competing interests

SSB and SW are authors of the original design and validation of the WEMWBS. The other authors have not declared any competing interests.

## Authors’ contributions

All authors contributed to, design, data collection and analysis and critically revised and approved the manuscript.

## Supplementary Material

Additional file 1: Table S1a WEMWBS scores across demographic groups for quantitative evaluation in Coventry.**Table S1b.** WEMWBS scores across demographic groups for quantitative evaluation in Birmingham. **Table S2.** Demographic description of focus group participants. Click here for file
