# Supplementary material for: Cross cultural evaluation of the Warwick-Edinburgh mental well-being scale (WEMWBS) -a mixed methods study
Source: Health Qual Life Outcomes. 2013 Feb 27;11:27. doi: 10.1186/1477-7525-11-27 (PMC3610169; doi:10.1186/1477-7525-11-27)
Supplement: Additional file 1: Table S1a — WEMWBS scores across demographic groups for quantitative evaluation in Coventry.Table S1b. WEMWBS scores across demographic groups for quantitative evaluation in Birmingham. Table S2. Demographic description of focus group participants. [file 1477-7525-11-27-S1.pdf]

Table S1a WEMWBS scores across demographic groups for quantitative survey in Coventry

	Chinese		Pakistani	
**Variable**	**N**	**Mean (95% Confidence Interval)**	**N**	**Mean (95% Confidence Interval)**
All	41	52.34 (50.04 – 54.65)	82	52.00 (50.08 – 53.93)
Gender*				
Male	29	53.21 (50.44-55.97)	57	52.89 (50.50-55.29)
Female	12	50.25 (45.65-54.85)	24	49.75 (46.36-53.14)
**Age in years***				
16-24	24	53.12 (50.08-56.17)	20	53.15 (50.50-55.8)
25-49	10	49.50 (43.55-55.45)	33	51.52 (48.04-54.99)
50-75	1		7	51.57 (47.27-55.87)
76-90	1			
**Employment Status**				
In work	3	51.00 (33.61-68.39)	41	53.8 (50.77-56.84)
Full time student	29	52.86 (50.22-55.51)	14	52.93 (50.12-55.73)
No paid work	9	51.11 (44.17 – 58.06)	27	48.78 (45.49-52.06)

**Marital Status***				
Single	34	51.68 (49.03-54.33)	23	53.26 (50.91-55.61)
Married	6	55.00 (49.69-60.31)	54	51.52 (48.87-54.17)
Other	1		2	57.50 (-37.8-152.8)
**Education**				
GCSE	1		4	56.50 (51.91-60.09)
5 GCSE	2	52.5 (-4.68-109.68)	12	47.50 (39.26-55.74)
A Level	14	52.86 (48.29-57.42)	18	53.83 (49.92-57.74)
Degree	12	52.08 (48.86-55.31)	20	54.10 (50.57-57.63)
Unknown	2	59.00 (-17.24 - 135.24)	7	54.86 (49.25-60.47)
None#	10	51.00 (44.27-57.73)	21	49.19 (45.53-52.85)

* Numbers of participants in some categories do not always add up to the total because of missing data

Includes “N/A” or question left out

Table S1b WEMWBS scores across demographic groups for questionnaire in Birmingham

	Chinese		Pakistani	
**Variable**	**N**	**Mean (95% Confidence Interval)**	**N**	**Mean (95% Confidence Interval)**
All	111	48.3 (46.36 – 50.24)	101	47.71 (45.98 – 49.45)
**Gender**				
Male	60	46.68 (44.29 – 49.07)	51	47.73 (45.22 – 50.24)
Female	51	50.20 (47.03 - 53.36)	50	47.70 (45.22 – 50.18)
**Age in years**				
16-24	28	46.89 (42.76 – 51.03)	27	50.0 (47.43 – 52.57)
25-49	61	48.16 (45.47 – 50. 85)	70	47.29 (45.03 – 49.55)
50-75	22	50.45 (46.26 – 54.64)	4	39.75 (35.18 – 44.32)*
**In work**				
Paid work	76	49.37 (47.0 – 51.74)	55	46.96 (44.60 – 49.33)
Student	16	51.50 (46.82 – 56.18)	16	51.5 (47.48 – 55.53)
No paid work	19	41.32 (37.22 – 45.41)*	30	47.07 (43.63 – 50.51)
Born in UK	63	47.71 (45.10 – 50.32)	55	48.55 (46.32 – 50.78)
Born in another country	48	49.06 (46.06 - 52.06)	46	46.72 (43.93 – 49.51)

Table S2 Demographic description of focus group participants

	**Chinese N (men, women)**	**Pakistani N (men, women)**
**All**	22 (8,14)	47(28,19)
**Age in years**		
16-24	6 (1,5)	17 (8,9)
25-49	9 (5,4)	20 (10,10)
50-75	7 (2, 5)	10 (10,0)
**Multiple deprivation index** based on postcode.<br><br>Number in most deprived 10% in England	12 (54%)	43 (91%)
**Terminal Education Age**		
15 or less	3 (1,2)	9 (2, 7)
16 - 20	4(1,3)	21 (11, 10)
21 or more	12(5,7)	8 (8, 0)
Still studying	3 (1, 2)	9 (7, 2)
**Employment**		
Paid work	16 (7, 9)	20 (17, 3)
Student	3 (1,2)	9 (7, 2)
No paid work	3(0, 3)	18 (4, 14)
